# Urine Metabolomics Analysis in Patients With Normoalbuminuric Diabetic Kidney Disease

**DOI:** 10.3389/fphys.2020.578799

**Published:** 2020-10-06

**Authors:** Qian Feng, Yuanmeng Li, Yuwei Yang, Jiafu Feng

**Affiliations:** ^1^College of Medical Technology, Chengdu University of Traditional Chinese Medicine, Chengdu, China; ^2^Department of Medical Laboratory, Affiliated Hospital of Southwest Medical University, Luzhou, China; ^3^Department of Clinical Laboratory, Mianyang Central Hospital, Mianyang, China

**Keywords:** diabetic kidney disease, diabetes mellitus, glomerular filtration rate, metabolomics, albuminuria

## Abstract

**Objective:**

Diabetic kidney disease (DKD) leads to low high albuminuria and gradually progresses to very high albuminuria with kidney insufficiency. However, about 20–40% of DKD is normoalbuminuric DKD (NADKD), which has impaired kidney function but normal urine albumin. This study is to investigate the urine metabolomic profiles of patients with NADKD and albuminuria DKD (ADKD).

**Methods:**

In total, 95 patients were divided into a simple diabetes mellitus group (SDM group), an ADKD group, and a NADKD group. All subjects were analyzed for urine metabolites using non-targeted metabolomics based on ultra-performance liquid chromatography – tandem mass spectrometry.

**Results:**

The urine metabolomic profiles of the SDM group, NADKD group, and ADKD group were significantly different, and 65 different metabolites were identified among the three groups. Metabolic pathway analysis of these differential metabolites found that the top three significantly changed metabolic pathways were linoleic acid metabolism, citrate cycle, and, arginine and proline metabolism. There are 12 metabolites enriched in these three metabolic pathways. In detail, compared with those in the SDM group, the levels of γ-linolenic acid in the ADKD group were increased significantly, while the levels of succinic acid, *cis*-aconitic acid, citric acid, L-proline, L-erythro-4-hydroxyglutamate, *N*-methylhydantoin, *N*-carbamoylputrescine, spermidine, and 5-aminopentanoic acid were reduced significantly; compared with those in the NADKD group, the levels of linoleic acid, γ-linolenic acid, and L-malic acid in the ADKD group were increased significantly (*P* < 0.05), while the levels of L-proline, L-erythro-4-hydroxyglutamate, *N*-carbamoylputrescine, and spermidine were significantly reduced (*P* < 0.05). However, there were no significant difference between the SDM group and NADKD group (*P* > 0.05).

**Conclusion:**

The urine metabolomic profiles between the NADKD group and the ADKD group are significantly different. Specifically, these two groups have distinct levels of linoleic acid, γ-linolenic acid, L-malic acid, L-proline, L-erythro-4-hydroxyglutamate, *N*-carbamoylputrescine, and spermidine.

## Introduction

Diabetic kidney disease (DKD) is the most common microvascular complication in patients with diabetes mellitus (DM). Approximately 20–40% of DM patients worldwide will develop DKD (American Diabetes Association, 2019b; Akin and Boluk, 2020). Persistent albuminuria [urine albumin – creatinine ratio (UACR) > 30 mg/g] is one of the clinical characteristics of DKD patients and also one of the laboratory indicators used in the clinical diagnosis of DKD. However, recent studies (Pichaiwong et al., 2019; Viswanathan et al., 2019) have shown that although many patients with DM have kidney glomerular filtration dysfunction after kidney injury, they have normal albuminuria. This is called normoalbuminuric DKD (NADKD) (Gohda et al., 2018).

There is a significant distinction in physiopathology between NADKD patients and albuminuria DKD (ADKD) patients. First of all, NADKD patients are generally female and older (Lamacchia et al., 2018; Penno et al., 2018). Secondly, NADKD patients are prone to large vessel damage and atherosclerosis of the kidney (Ekinci et al., 2013; Penno et al., 2018). Therefore, some studies speculate that the occurrence of NADKD may be related to genetic factors, estrogen levels, and kidney injury patterns in patients with NADKD (Chen et al., 2017; Silva et al., 2017; Yamanouchi et al., 2019). However, prospective studies exploring the pathogenesis of NADKD at the metabolomics level are limited.

Herein, we used ultra-performance liquid chromatography – tandem mass spectrometry (UPLC–MS/MS) to analyze the metabolomic profiles of NADKD and ADKD patients. Their relationship with commonly used renal function indicators was also analyzed. Our findings may provide insights for the study of the pathogenesis of NADKD and the screening of biomarkers for NADKD.

## Materials and Methods

### Ethics Statement

This study was reviewed and approved by the Medical Ethics Committee of Mianyang Central Hospital (Approval Nos. S2014048 and S2018085), and all patients signed informed consent.

### Patients

The PASS 11.0.7 software (NCSS, United States) was used to calculate the sample size and perform power analysis. When there are three groups, 81 cases are needed for *Correlation Power Analysis*, and 27 cases are needed for *Multiple Comparisons*. Therefore, a total of 95 patients with DM who were treated at Mianyang Central Hospital from June to August 2019 were selected. There were 44 females, aged 31–85 (average: 61.73 ± 12.51 years; median: 62 years), and 51 males, aged 33–89 (average: 55.67 ± 12.09 years; median: 53 years). According to KDIGO 2012 clinical practice guidelines for the Evaluation and Management of CKD (National Kidney Foundation, 2013) and the levels of UACR and estimated glomerular filtration rate (eGFR) levels, DM patients were divided into three groups: (1) the SDM group (UACR < 30 mg/g and eGFR ≥ 90 ml/min/1.73 m^2^, *n* = 30 cases); (2) ADKD group (30 ≤ UACR < 300 mg/g and eGFR ≥ 45 ml/min/1.73 m^2^, *n* = 30 cases); (3) NADKD group (DKD patients with UACR < 30 mg/g and 45 ≤ eGFR < 90 ml/min/1.73 m^2^, *n* = 35 cases). Inclusion criteria included (1) age ≥ 18 years; (2) clinical diagnosis of DM, which was in line with the American Diabetes Association (ADA) criteria (American Diabetes Association, 2019b), and a history of DM greater than 5 years; (3) for the DM group, UACR ≤ 30 mg/g and eGFR ≥ 60 ml/min/1.73 m^2^; and (4) a diagnosis of early DKD that met the ADA criteria for DM microvascular complications (American Diabetes Association, 2019a). Exclusion criteria included (1) age <18 years; (2) history of DM less than 5 years; (3) primary or secondary kidney function injury caused by other reasons, such as rapid rise in UACR in the short term, rapid decline in eGFR, or non-DM diseases, such as interstitial kidney disease, kidney stones, and nephrotic syndrome; (4) suffering from diseases that affected urinary albumin secretion and eGFR, such as benign and malignant tumors, hypertension, and urogenital infections; (5) kidney transplantation; (6) menstruating, pregnant, and lactating women.

### Sample Collection

Venous blood (5.0 ml) was collected from each patient after overnight fasting. Serum was isolated after centrifugation at 3,000 *g* for 15 min. After blood sample collection, midstream urine sample of about 10.0 ml was collected. All samples were collected at approximately the same time each day (between 8 and 10 am every day).

### Measurement of Common Kidney Function Indicators

The levels of serum creatinine (SCr), cystatin C (CysC), and complement C1q (C1q) were detected using a LABOSPECT 008 AS automatic biochemical analyzer (Hitachi, Japan). The detection methods were the sarcosine oxidase method for SCr and transmission turbidimetry for CysC and C1q. The eGFR was calculated using the eGFR formula developed by our laboratory based on the Chinese population (Feng et al., 2013), i.e., eGFR (ml/min/1.73 min) = 78.64 × CysC (mg/L)^–0.964^.

The urinary albumin and urine creatinine levels were measured on an A25 automatic specific protein analyzer (BioSystems, Spain) using transmission immunoturbidimetry and the sarcosine oxidase method, respectively. The UACR was calculated as UACR (mg/g) = urinary albumin (mg/L)/urine creatinine (g/L).

### UPLC–MS/MS

The urine sample was mixed with 80% methanol, vortexed, and centrifuged at 10,000 *g* at 4°C for 10 min. The supernatant was collected, filtered using a 0.22 μm filter membrane, and then analyzed by UPLC–MS/MS. An UltiMate 3000 high-performance liquid chromatograph (Thermo, United States) was used for chromatographic analysis. The chromatographic conditions were as follows. Chromatographic separation was performed using an ACQUITY UPLC^®^ HSS T3 (1.8 μm, 2.1 × 150 mm) column (Waters, United States). The column temperature was 40°C. The flow rate was 0.25 ml/min. In positive ion mode, mobile phase A was an aqueous solution containing 0.1% formic acid (TCI, Japan), and mobile phase B was an acetonitrile (Thermo, United States) solution containing 0.1% formic acid; in negative ion mode, mobile phase A was 5 mmol/L of aqueous solution of ammonium formate (Sigma, United States), and mobile phase B was 100% acetonitrile. The gradient elution conditions were as follows: 0–1 min, 98% mobile phase A; 1–9 min, 98–50% mobile phase A; 9–12 min, 50–2% mobile phase A; 12–13.5 min, 2% mobile phase A; 13.5–14 min, 2–98% mobile phase A; 14–17 min, 98% mobile phase A (positive ion mode); 14–20 min, 98% mobile phase A (negative ion mode). The reagents were MS grade. A Q Exactive Focus mass spectrometer (Thermo, United States) was used for MS analysis with an ESI ion source. The MS conditions were as follows: positive ion spray voltage 3.5 kV, negative ion spray voltage 2.5 kV, sheath gas 30 arb and auxiliary gas 10 arb. Full scan was performed with a resolution of 70,000, and the scan range was 81–1,000 Da. The secondary cracking was carried out by high-energy-induced cracking with a collision voltage of 30 eV.

ProteoWizard software was used to convert the raw data into mzXML format. The XCMS package was used for peak detection and normalization. Multivariate statistical analysis was performed using R. Language ropls package. The metabolites were identified using databases of Metlin^1^, MoNA^2^, and HMDB^3^ and verified the metabolites using the BioDeep metabolome database (BioNovoGene; Suzhou, China). MetaboAnalyst^4^ was used for the analysis of metabolic pathways.

### Statistical Analysis

SPSS 25.0 was used for statistical analysis. Data of normal distribution are expressed as mean ± SD. If there is homogeneity of variance, ANOVA was used for comparisons among multiple groups followed by LSD *t*-test; if not, Welch’s *t*-test was performed for comparisons among multiple groups followed by Dunnet’s T3 test. Measurement data of non-normal distribution are expressed as median (interquartile range) [M (P25, P75)]. Differences between groups were compared using the independent-sample Kruskal–Wallis test. Comparisons of count data among groups were compared using chi-square test. Spearman’s correlation was used for correlation analysis. *P* < 0.05 was considered statistically significant.

## Results

### Clinical Data of Patients

The clinical data comparison of patients is shown in Table 1. Among all factors, only age showed a normal distribution, and the remaining factors, including UACR, eGFR, SCr, CysC, and C1q, were non-normally distributed. Statistically, there was no significant difference in sex and C1q among SDM, NADKD, and ADKD (*P* > 0.05). However, age, UACR, eGFR, SCr, and CysC had statistical differences among the groups (*P* < 0.05). Pairwise comparison analysis showed that compared with the SDM group, the age and CysC of the NADKD group increased while eGFR decreased (*P* < 0.05). Compared with the SDM group, the age, UACR, SCr, and CysC of the ADKD group increased, while eGFR decreases (*P* < 0.05). Compared with the NADKD group, the ADKD group had a significant increase in UACR (*P* < 0.05). There was no significant difference between the NADKD group and ADKD group in the common serum kidney function indicators such as SCr, CysC, C1q, and eGFR.

**TABLE 1:** Clinical data of subjects (*n* = 95).

**Group**	**SDM (*n* = 30)**	**NADKD (*n* = 30)**	**ADKD (*n* = 35)**	**χ^2^/*F***	***P***
Male/female (*n*)	14/16	18/12	19/16	1.081	0.583
Age (year)	51.57 ± 1.763	61.87 ± 2.121^△^	61.49 ± 2.282^△^	9.188	0.000
UACR (mg/g)	15.585 (8.63, 19.91)	10.79 (8.14, 20.75)	92.10 (69.89, 144.53)^△▲^	65.787	0.000
eGFR (ml/min/1.73 min)	98.70 (93.84, 105.47)	76.43 (60.07, 87.99)^△^	78.64 (56.86, 86.12)^△^	43.288	0.000
SCr (μmol/L)	55.95 (45.27, 70.68)	73.70 (62.92, 82.25)^△^	72.90 (55.10, 84.90)^△^	15.778	0.000
CysC (mg/L)	0.79 (0.74, 0.83)	1.03 (0.89, 1.32)^△^	1.00 (0.91, 1.40)^△^	42.910	0.000
C1q (mg/L)	218.50 (192.25, 246.98)	197.50 (170.25, 214.50)	205.00 (190.00, 233.00)	5.822	0.054

*SDM, simple diabetes mellitus; NADKD, normoalbuminuric diabetic kidney disease; ADKD, albuminuria diabetic kidney disease; UACR, urine albumin/creatinine ratio; eGFR, estimated glomerular filtration rate; SCr, serum creatinine; CysC, serum cystatin C; C1q, serum complement C1q subunit. Compared with SDM group, ^△^ P < 0.05. Compared with NADKD group, ^▲^P < 0.05.*

### Multivariate Statistical Analysis of Metabolites

**FIGURE 1:**
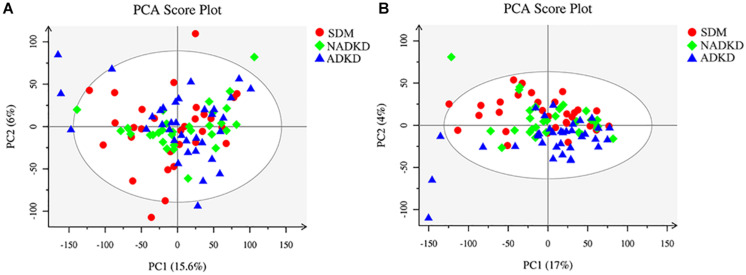
Plots of PCA score. **(A)** PCA score plot in positive ion mode. **(B)** PCA score plot in negative ion mode.

The UPLC-MS/MS method was used to detect all samples in positive and negative ion modes, respectively, and the processed data were subjected to multivariate statistical analysis. We showed the original state of all sample data through the PCA score plot. From the results of the PCA score plot, the sample was basically within the Hotelling *T*-squared ellipse, and the urine components of the SDM group, NADKD group, and ADKD group did not achieve effective separation (Figures 1A,B).

**FIGURE 2:**
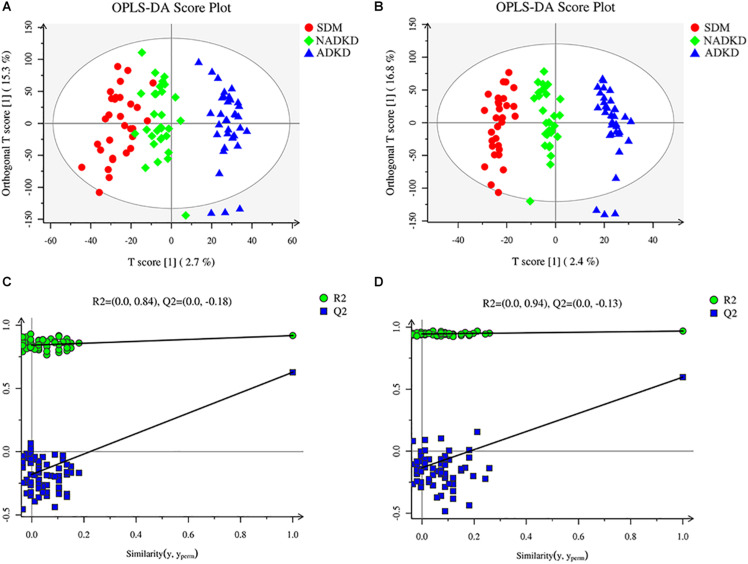
Plots of OPLS-DA score and permutation test. **(A)** OPLS-DA score plot in positive ion mode. **(B)** OPLS-DA score plot in negative ion mode. **(C)** OPLS-DA permutation test plot in positive ion mode. **(D)** OPLS-DA permutation test plot in negative ion mode. The criterion for evaluating whether there is overfitting in the OPLS-DA model is that the regression line at a blue Q2 point crosses or is less than 0 from the abscissa.

To screen the significantly differential metabolites, we performed orthogonal partial least squares-discriminant analysis (OPLS-DA). The results showed that the SDM group, NADKD group, and ADKD group could be separated in the positive and negative modes (Figures 2A,B). The permutation test was performed to evaluate whether there is overfitting in the OPLS-DA models. In total, 100 random permutation tests were carried out by R package ropls, which showed that there was no overfitting (Figures 2C,D). The results showed that there were differences in metabolic profiles among the SDM, NADKD, and ADKD groups.

### Differential Metabolite Screening

**FIGURE 3:**
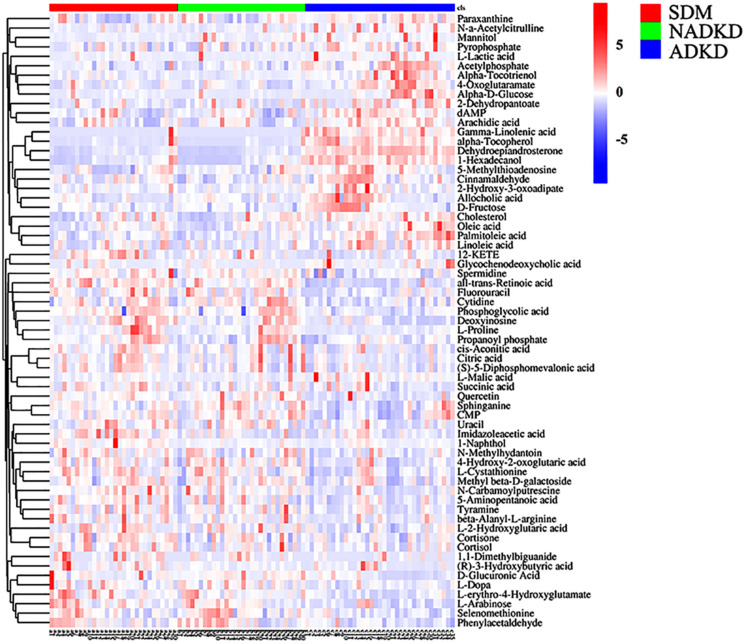
Differential metabolite heat map. The columns represent samples, the rows represent metabolites, and the relative content of the metabolites is displayed by color. The heat map shows differential metabolites among SDM, NADKD, and ADKD groups.

Based on the analysis results of OPLS-DA and using the variable importance for the projection (VIP) >1 and *P* < 0.05 as the screening criteria, we screened out the differential metabolites using the Metlin, MoNA, and BioDeep metabolome databases. We found a total of 65 differential metabolites in the SDM, NADKD, and ADKD groups, which were displayed as a heat map (Figure 3).

### Analysis of Metabolic Pathways

**FIGURE 4:**
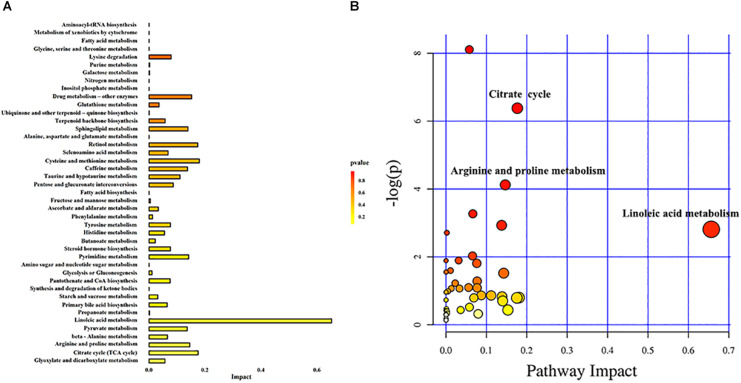
Metabolic pathway analysis. **(A)** Histogram of metabolic pathways. **(B)** Bubble diagram of metabolic pathways. Each bubble in the bubble diagram represents a metabolic pathway. Color gradient and circle size indicate the significance of the pathway ranked by *P*-value (yellow: higher *P*-values and red: lower *P*-values) and pathway impact score (the larger the circle, the higher the pathway impact score). According to the −log(*P*) value and pathway impact score, the top three metabolic pathways were identified by name.

In order to understand the metabolic pathways that may be involved in the occurrence of DKD, we used the MetaboAnalyst database to perform pathway enrichment and topological analysis of differential metabolites in urine. We screened out 43 metabolic pathways (Figure 4A). According to the −log(*P*) value and pathway impact score, the top three metabolic pathways were selected, which were linoleic acid metabolism, citrate cycle, and arginine and proline metabolism (Figure 4B). Meanwhile, the metabolic pathway analysis results also showed that there were 12 different metabolites enriched in these three metabolic pathways, which were linoleic acid and γ-linolenic acid of linoleic acid metabolism; succinic acid, L-malic acid, *cis*-aconitic acid, and citric acid of citrate cycle; and L-proline, L-erythro-4-hydroxyglutamate, *N*-methylhydantoin, *N*-carbamoylputrescine, spermidine, and 5-aminopentanoic acid of arginine and proline metabolism (Table 2).

**TABLE 2:** Analysis of the top three metabolic pathways.

**Name**	**−Log(*P*)**	**Impact**	**Compounds**	**Pathway**
Linoleic acid metabolism	2.811	0.656	Linoleic acid and γ-linolenic acid	hsa00591
Citrate cycle	6.380	0.176	Succinic acid, L-malic acid, *cis*-aconitic acid, and citric acid	hsa00020
Arginine and proline metabolism	4.122	0.147	L-Proline, L-erythro-4-hydroxyglutamate, *N*-methylhydantoin, *N*-carbamoylputrescine, spermidine, and 5-aminopentanoic acid	hsa00330

### Differential Metabolite Analysis

**FIGURE 5:**
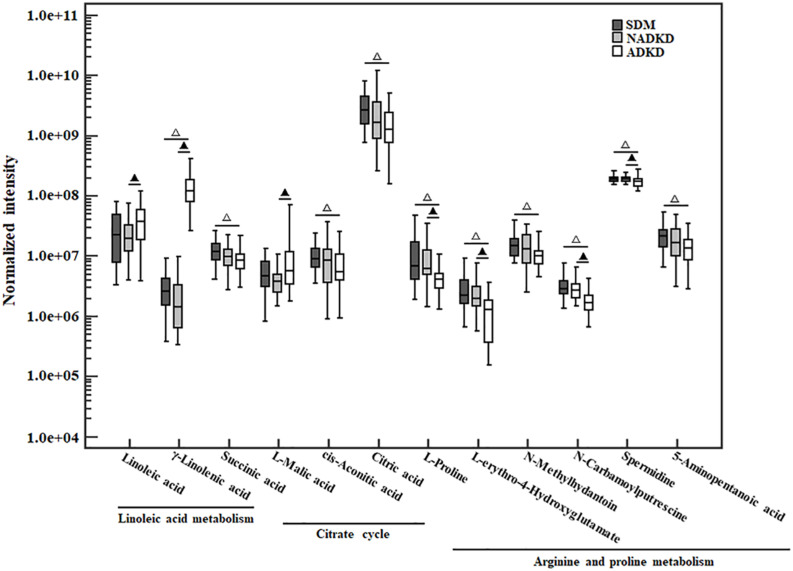
The box plot of normalized intensity peak areas of differential metabolites. Compared with the SDM group, △*P* < 0.05. Compared with the NADKD group, ▲*P* < 0.05.

The non-parametric test was used to analyze the differential metabolites enriched in linoleic acid metabolism, citrate cycle, and arginine and proline metabolism pathways. Compared with those in the SDM group, the levels of linoleic acid metabolism-related metabolites γ-linoleic acid (*F* = −40.433, *P* = 0.000) were increased significantly in the ADKD group, while the levels of citrate metabolism (citrate cycle)-related metabolites, including succinic acid (*F* = 18.019, *P* = 0.026), *cis*-aconitic acid (*F* = 17.981, *P* = 0.026), and citric acid (*F* = 22.395, *P* = 0.003), and arginine and proline metabolism-related metabolites, including L-proline (*F* = 24.886, *P* = 0.001), L-erythro-4-hydroxyglutamate (*F* = 29.643, *P* = 0.000), *N*-methylhydantoin (*F* = 19.562, *P* = 0.013), *N*-carbamoylputrescine (*F* = 30.943, *P* = 0.000), spermidine (*F* = 16.971, *P* = 0.040), and 5-aminopentanoic acid (*F* = 19.262, *P* = 0.015) were reduced significantly in the ADKD group. Compared with those in the NADKD group, the levels of linoleic acid metabolism-related metabolites of linoleic acid (*F* = −16.414, *P* = 0.046) and γ-linolenic acid (*F* = −46.967, *P* = 0.000) and the citrate metabolism-related metabolite of L-malic acid (*F* = −18.438, *P* = 0.022) was increased significantly in the ADKD group, while the levels of arginine and proline metabolism-related metabolites of L-proline (*F* = 24.152, *P* = 0.001), L-erythro-4-hydroxyglutamate (*F* = 22.743, *P* = 0.003), *N*-carbamoylputrescine (*F* = 24.610, *P* = 0.001), and spermidine (*F* = 16.505, *P* = 0.048) were reduced significantly in the ADKD group. However, there was no significant difference in the levels of these metabolites between the SDM group and the NADKD group (Figure 5).

### Correlation Analysis of Different Metabolites and Kidney Function Indicators

Spearman correlation was used to analyze the correlation of linoleic acid, γ-linolenic acid, succinic acid, L-malic acid, *cis*-aconitic acid, citric acid, L-proline, L-erythro-4-hydroxyglutamate, *N*-methylhydantoin, *N*-carbamoylputrescine, spermidine, and 5-aminopentanoic acid with common kidney function indicators. As shown in Table 3, eGFR was significantly positively correlated with succinic acid, *cis*-aconitic acid, citric acid, L-erythro-4-hydroxyglutamate, *N*-methylhydantoin, *N*-carbamoylputrescine, and 5-aminopentanoic acid, while significantly negatively correlated with γ-linolenic acid. CysC was significantly positively correlated with γ-linolenic acid, whereas significantly negatively correlated with succinic acid, *cis*-aconitic, citric acid, L-erythro-4-hydroxyglutamate, *N*-methylhydantoin, *N*-carbamoylputrescine, and 5-aminopentanoic acid. SCr was significantly negatively correlated with succinic acid, *cis*-aconitic, citric acid, and 5-aminopentanoic acid. C1q was significantly positively correlated with γ-linolenic acid, succinic acid, and L-malic acid. UACR was significantly positively correlated with linoleic acid, γ-linolenic acid, L-malic acid, while being significantly negatively correlated with L-erythro-4-hydroxyglutamate, *N*-carbamoylputrescine, spermidine, and 5-aminopentanoic acid. Additionally, *cis*-aconitic acid had the strongest correlation with eGFR (*r* = 0.499, *P* = 0.000) and CysC (*r* = −0.501, *P* = 0.000); succinic acid had the strongest correlation with SCr (*r* = 0.296, *P* = 0.000) and C1q (*r* = 0.265, *P* = 0.009); and γ-linolenic acid had the strongest correlation with UACR (*r* = 0.812, *P* = 0.000).

**TABLE 3:** Correlation analysis of differential metabolites and kidney function indicators.

**Indicator**	**Statistic**	**eGFR**	**CysC**	**SCr**	**C1q**	**UACR**
Linoleic acid	*r*	0.055	−0.056	0.007	0.035	0.328
	*P*	0.598	0.592	0.943	0.738	0.001
γ-Linolenic acid	*r*	−0.228	0.229	0.126	0.202	0.812
	*P*	0.026	0.026	0.222	0.050	0.000
Succinic acid	*r*	0.335	−0.336	−0.296	0.265	−0.158
	*P*	0.001	0.001	0.004	0.009	0.126
L-Malic acid	*r*	0.049	−0.048	−0.031	0.257	0.299
	*P*	0.638	0.643	0.766	0.012	0.003
*cis*-Aconitic acid	*r*	0.499	−0.501	−0.231	0.163	−0.150
	*P*	0.000	0.000	0.024	0.115	0.147
Citric acid	*r*	0.413	−0.413	−0.257	0.063	−0.174
	*P*	0.000	0.000	0.012	0.543	0.091
L-Proline	*r*	0.038	−0.039	−0.048	−0.092	−0.179
	*P*	0.715	0.708	0.642	0.376	0.083
L-Erythro-4-hydroxyglutamate	*r*	0.232	−0.234	−0.170	−0.061	−0.471
	*P*	0.024	0.022	0.100	0.556	0.000
*N*-Methylhydantoin	*r*	0.241	−0.236	−0.060	0.131	−0.194
	*P*	0.019	0.021	0.561	0.206	0.060
*N*-Carbamoylputrescine	*r*	0.253	−0.251	−0.125	0.155	−0.367
	*P*	0.013	0.014	0.226	0.134	0.000
Spermidine	*r*	−0.061	0.058	−0.112	−0.036	−0.231
	*P*	0.557	0.577	0.281	0.730	0.025
5-Aminopentanoic acid	*r*	0.424	−0.424	−0.243	0.064	−0.222
	*P*	0.000	0.000	0.018	0.540	0.031

*UACR, urine albumin/creatinine ratio; eGFR, estimated glomerular filtration rate; SCr, serum creatinine; CysC, serum cystatin C; C1q, serum complement C1q subunit.*

## Discussion

Metabolomics is a detection technique with the advantages of high resolution, high throughput, and high sensitivity. According to different research purposes, metabolomics can be divided into targeted and non-targeted metabolomics analysis. Non-targeted metabolomics is based on limited related research and background knowledge. It can systematically and comprehensively analyze the metabolites, thus obtaining a large amount of metabolite data and identifying differential metabolites (Johnson et al., 2016). It has been widely used in the research of many diseases, such as lung cancer (Seow et al., 2019), pancreatic cancer (Shu et al., 2018), prostate cancer (Jayaraman et al., 2018), heart disease (Li et al., 2016), and liver disease (Dong et al., 2017). Recently, the metabolomics related to the occurrence and development of DKD has attracted much attention (Rhee, 2015). Although there have been some reports on metabolomics in DKD patients, these studies (Chen et al., 2018; Tang et al., 2019) have mostly focused on DKD patients with elevated urinary albumin. However, studies on NADKD patients have been rare.

This study used UPLC-MS/MS to analyze the urine metabolites of NADKD patients and ADKD patients. PCA results showed that the urine components of SDM, NADKD, and ADKD could not be effectively separated. We speculate that the main reason is that PCA is an unsupervised analysis method, which pays more attention to differences between groups. When the differences within groups are too large due to individual differences in subjects or daily diet and other factors, PCA cannot eliminate the differences within groups, leading to omission of differential metabolites between groups (Bradley and Robert, 2013). Therefore, we further performed OPLS-DA. The results showed that the SDM group and the NADKD group could be clearly distinguished from the ADKD group, indicating that the urine metabolites of the NADKD group and ADKD group are significantly different. There were 65 different metabolites with significant changes among the three groups. Subsequently, metabolic pathway analysis of these differential metabolites found that the top three metabolic pathways with significant changes were linoleic acid metabolism, citrate cycle, and arginine and proline metabolism and that there were 12 different metabolites enriched in these three metabolic pathways, including linoleic acid, γ-linolenic acid, succinic acid, L-malic acid, *cis*-aconitic acid, citric acid, L-proline, L-erythro-4-hydroxyglutamate, *N*-methylhydantoin, *N*-carbamoylputrescine, spermidine, and 5-aminopentanoic acid.

Mitochondrial dysfunction is one of the mechanisms that promote the occurrence and development of DKD (Yang et al., 2017). The levels of metabolites produced during the citrate cycle are mostly affected by the mitochondria. Studies have found that compared with DM without renal damage, the levels of metabolites such as citric acid, *cis*-aconitic acid, glycolic acid, and aconitic acid in the urine of DKD patients are significantly reduced, suggesting that DKD patients may have mitochondrial dysfunction (Sharma et al., 2013; Saulnier et al., 2018). In this study, we found that compared with the SDM group, the levels of succinic acid, *cis*-aconitic acid, and citric acid in the ADKD group were reduced significantly, which was consistent with previous reports (Sharma et al., 2013; Saulnier et al., 2018). However, no significant difference was found between the SDM group and the NADKD group. Compared with the ADKD group, only the level of L-malic acid was significantly increased in the NADKD group. Meanwhile, the correlation analysis showed that there was no correlation between L-malic acid and eGFR. Moreover, although L-malic acid had a positive correlation with UACR, it was a weak positive correlation. Thus, the changes in citrate cycle were different in the NADKD group and the ADKD group. We speculate that this may be related to the different pathogeneses of NADKD and ADKD.

L-Proline is a substrate for collagen synthesis and can be metabolized *in vivo* by L-arginine. It participates in tissue repair with L-arginine and other metabolites. L-Proline and its analogs can reduce kidney injury caused by toxins or DM by reducing oxidative stress (Kumari et al., 2016; Li et al., 2019). In this study, we showed that compared with the SDM group, the levels of arginine and proline metabolism-related metabolites, including L-proline, L-erythro-4-hydroxyglutamate, *N*-methylhydantoin, *N*-carbamoyl putrescine, spermidine, and 5-aminopentanoic acid, in the ADKD group were significantly reduced. Compared with the NADKD group, the levels of L-proline, L-erythro-4-hydroxyglutamate, and spermidine in the ADKD group were significantly reduced. Thus, compared with those in the SDM group and the NADKD group, the levels of most metabolites related to arginine and proline metabolism in the urine of the ADKD group were decreased significantly or showed a downward trend. In addition, except for L-proline, the other five arginine and proline metabolism-related metabolites were negatively correlated with UACR; and except for L-proline and spermidine, the other four arginine and proline metabolism-related metabolites were positively correlated with eGFR, while being negatively correlated with CysC. This suggests that metabolites related to arginine and proline metabolism have a certain correlation with renal function. At present, studies have reported that patients with DKD or non-DM kidney diseases have abnormal metabolism of arginine and proline (Sangeeta and Pradeep, 2016; Abbiss et al., 2019); however, there is no report on the correlation between arginine and proline metabolism-related metabolites and NADKD. Whether there is a relationship between the metabolism changes of arginine and proline and the occurrence of NADKD and whether they are markers of NADKD still need further study.

Linoleic acid and linolenic acid are essential polyunsaturated fatty acids with anti-inflammatory properties. Proper intake of these polyunsaturated fatty acids plays an active role in improving kidney function in patients with DKD (Garman et al., 2009; Mali et al., 2016; Dos Santos et al., 2018). However, it is shown that the levels of free fatty acids such as linoleic acid and arachidonic acid in the urine of patients with DKD are significantly increased (Sasaki et al., 2009). Under normal circumstances, β-oxidation of fatty acids is the main source of energy for proximal renal tubular cells. Free fatty acids can bind to albumin, filtered by the glomerulus, and reabsorbed in the renal tubules (Xu et al., 2015; Khan et al., 2018). When DKD patients suffer from kidney damage, as the excretion of urinary albumin increases, the fatty acids filtered by the glomerulus also increase, resulting in excessive free fatty acids bound to albumin being overloaded and reabsorbed in the renal tubules (Kamijo et al., 2002; Cobbs et al., 2018). This will cause severe tubular interstitial damage. The results of this study showed that compared with those in the SDM group, the levels of UACR and γ-linolenic acid in the ADKD group were significantly increased, which was similar to the results of previous studies (Kamijo et al., 2002; Sasaki et al., 2009). However, compared with those in the ADKD group, the levels of UACR, linoleic acid, and γ-linolenic acid in the NADKD group were reduced. And there was no significant difference between the SDM group and NADKD group in the levels of UACR, linoleic acid, and γ-linolenic acid. It has been reported that the level of free fatty acids in urine can indirectly reflect renal tubular interstitial damage and is positively correlated with the degree of tubulointerstitial damage (Sasaki et al., 2009; Khan et al., 2018). Moreover, studies have found that perhaps due to the different pathogenesis of ADKD and NADKD, the degree of renal tubular damage in patients with NADKD is less severe than that of ADKD (Budhiraja et al., 2013). Thus, in this study, compared with the SDM group, the ADKD group and the NADKD group had different changes in the levels of linoleic acid and γ-linolenic acid; specifically, compared with those in the ADKD group, the levels of linoleic acid and γ-linolenic acid were significantly reduced, which may be related to the different pathogenesis of the NADKD and ADKD, and to confirm whether this is also related to the degree of damage to the renal tubules or renal interstitium of the two groups requires research. In addition, correlation analysis also showed a significant positive correlation of linoleic acid and γ-linolenic acid with UACR, and γ-linolenic acid was also the metabolite with the strongest correlation with UACR among the 12 metabolites. It is suggested that the UACR levels of DKD patients are related to their urine linoleic acid and γ-linolenic acid levels. However, the underlying mechanism still needs further study.

This study found that although there were no significant differences in the levels of eGFR, SCr, C1q, and CysC between patients with NADKD and ADKD, metabolomics analysis found a significant difference in urine metabolites between patients with NADKD and ADKD. NADKD patients were significantly different from ADKD patients in linoleic acid, γ-linolenic acid, L-malic acid, L-proline, L-erythro-4-hydroxyglutamate, *N*-carbamoylputrescine, and spermidine. However, no significant differences were found in these metabolites between the SDM and NADKD groups. This may be caused by a variety of reasons. First, urine metabolomics is inevitably affected by factors such as the subject’s age, diet, medication, and sample size. Second, it is found that UACR and GFR were normal in some patients with DM; however, kidney biopsy showed that kidney structure changes in these patients were similar to those of DKD patients (Comai et al., 2019). In this study, we did not perform a histopathological examination on all subjects. Therefore, there may be undetected NADKD or ADKD patients in the SDM group. This may cause the non-significant difference in the metabolomics between the SDM group and the NADKD group. In addition, metabolic pathway analysis also showed differences in linoleic acid metabolism, citrate cycle, and arginine and proline metabolism among the three groups of subjects. However, whether these differential metabolites and their metabolic pathways are involved in the pathogenesis of NADKD requires further investigation. In the future, we will conduct multicenter studies and more comprehensive metabolomics analysis on NADKD patients, in order to understand the pathogenesis of NADKD and identify potential biomarkers for NADKD diagnosis. In addition, according to previous studies (Lamacchia et al., 2018; Penno et al., 2018), NADKD patients are mostly female. However, there was no sex difference in the included subjects of this study. This may be caused by the different populations involved. Further studies are needed to explain this.

In conclusion, we found that ADKD patients were significantly different from SDM and NADKD patients in the levels of metabolites related to the linoleic acid metabolism, citrate cycle, and arginine and proline metabolism. However, no significant difference was found in these metabolites between the SDM and NADKD groups. In this study, through UPLC–MS/MS-based metabolomics analysis, we found that there were significant differences in urine metabolic profiles between NADKD and ADKD patients, which may be due to the different pathogeneses of NADKD and ADKD. Additionally, we also confirmed that UPLC–MS/MS-based metabolomics analysis has potential to demonstrate the pathogenesis of NADKD and identify its diagnostic markers.

## Data Availability Statement

The data analyzed in this study is subject to the following licenses/restrictions: The data sets generated and/or analyzed during the current study are available from the corresponding author upon reasonable request. Requests to access these datasets should be directed to jiafufeng@foxmail.com.

## Ethics Statement

The studies involving human participants were reviewed and approved by the Medical Ethics Committee of Mianyang Central Hospital. The patients/participants provided their written informed consent to participate in this study.

## Author Contributions

QF and JF conceived the study. QF, YY, and YL curated the data and wrote the original draft of the manuscript. YL and YY participated in investigation. QF contributed to methodology. YL collected resources. JF supervised the study and wrote, reviewed and edited the manuscript. QF and YY contributed to visualization. All authors contributed to the article and approved the submitted version.

## Conflict of Interest

The authors declare that the research was conducted in the absence of any commercial or financial relationships that could be construed as a potential conflict of interest.

## Footnotes

1 http://metlin.scripps.edu

2 https://mona.fiehnlab.ucdavis.edu/

3 http://www.hmdb.ca/metabolites

4 http://www.Metaboanalyst.ca
